# Retraction note: Triptolide contributes to decrease in TLR4 expression by upregulating miR-224-3p to inhibit the inflammatory reaction in diabetic nephropathy

**DOI:** 10.18632/oncotarget.14017

**Published:** 2016-12-19

**Authors:** Bei Sun, Shaolan Hu, Yongmei Li, Endong Zhu, Li Li, Fei Han, Chun-Jun Li, Liming Chen

**Retraction**: Due to an error, Fig1 was previously published in another paper. The original source was never cited. The authors sincerely apologize for this error..

**Footnotes**: The online version of the original article can be found under doi.

Original article: Oncotarget. 2016 Nov 4. doi: 10.18632/oncotarget.13127.

